# A Case Report of Mycophenolate Mofetil-Induced Fulminant Colitis in a 76-Year-Old Renal Transplant Recipient

**DOI:** 10.7759/cureus.76505

**Published:** 2024-12-28

**Authors:** Tamiru B Berake, Mohammad Almeqdadi, Lense Negash, Saltanat Ualiyeva

**Affiliations:** 1 Internal Medicine, Tufts Medical Center, Boston, USA; 2 Gastroenterology and Hepatology, Tufts Medical Center, Boston, USA; 3 Internal Medicine, New York Medical College/Metropolitan Hospital, New York, USA; 4 Pathology and Laboratory Medicine, Tufts Medical Center, Boston, USA

**Keywords:** bowel perforation, fulminant colitis, inflammatory bowel disease, mycophenolate mofetil (mmf), pan-colitis

## Abstract

Mycophenolate mofetil (MMF) is a widely utilized immunosuppressive medication to prevent organ rejection in transplant recipients and manage autoimmune diseases. While gastrointestinal side effects, such as diarrhea and abdominal discomfort, are common, fulminant colitis is a rare complication. This case report describes the occurrence of fulminant colitis in a 76-year-old renal transplant recipient.

## Introduction

Mycophenolate mofetil (MMF) is a commonly used immunosuppressive medication, primarily prescribed to prevent organ rejection in transplant recipients and to treat various autoimmune disorders. While gastrointestinal side effects, such as bowel habit change, nausea, and vomiting, are quite common, MMF-induced colitis is a relatively rare occurrence that can mimic the clinical and histopathological features of inflammatory bowel disease [1]. Fulminant colitis, a severe and potentially life-threatening manifestation of MMF-induced gastrointestinal toxicity, presents significant diagnostic and therapeutic challenges. This case reports a complex presentation of fulminant colitis in a 76-year-old kidney transplant recipient who ultimately died as his condition was unresponsive to standard treatment approaches.

## Case presentation

**Figure 1:**
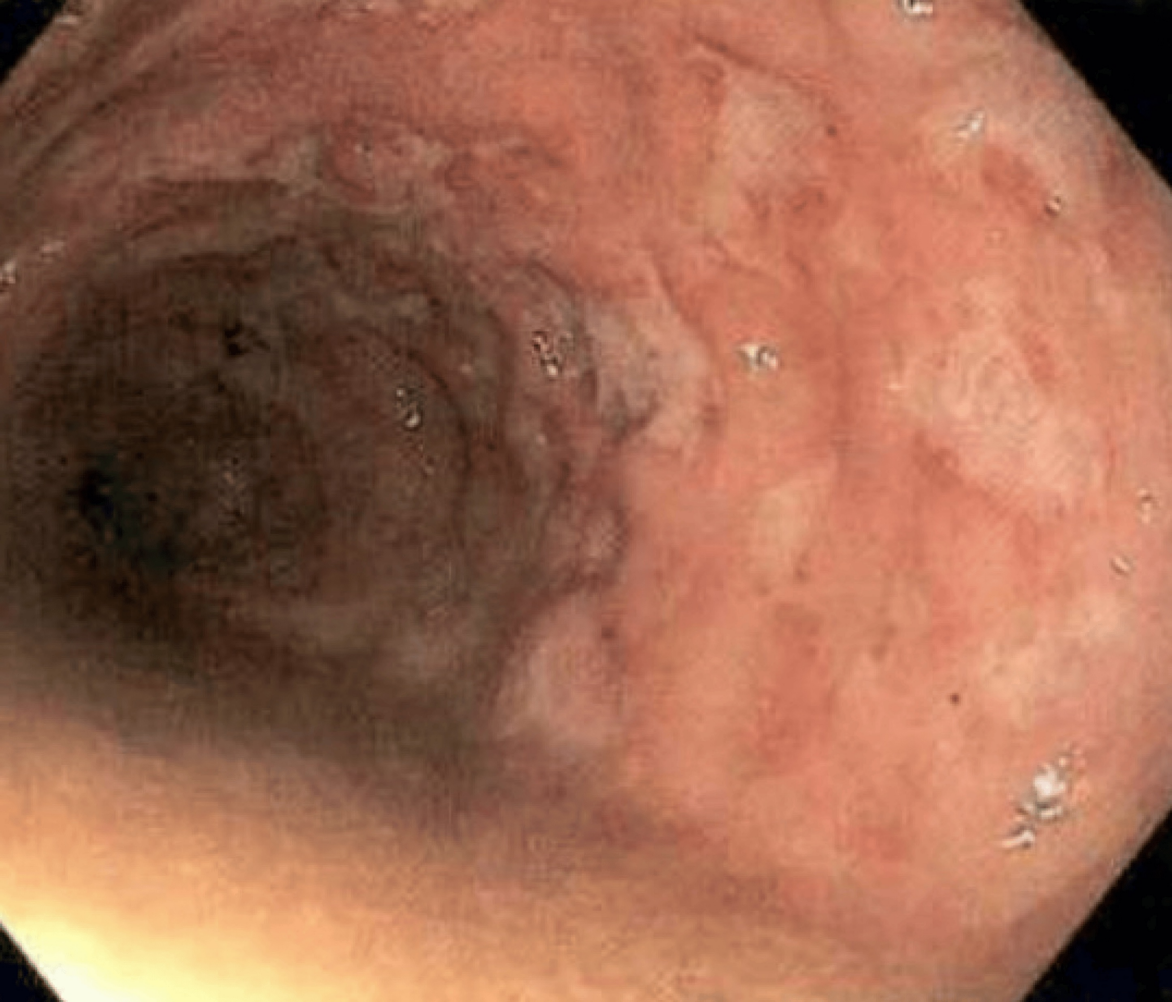
Colonoscopy. Colonoscopy shows multiple punched-out ulcers throughout the colon with skip lesion sparing terminal ileum.

A 76-year-old male with a medical history of hypertension, diabetes mellitus, and a deceased donor kidney transplant in 2017 with baseline creatinine of 1.0 maintained on tacrolimus and mycophenolate mofetil (MMF). He presented with a four-month history of watery diarrhea and progressive weight loss. Physical examination revealed abdominal tenderness without guarding or rebound tenderness. An extensive infectious workup was conducted, which included tests for CMV, giardiasis, cryptosporidium, and HIV, all of which were negative. Workup for chronic diarrhea for chronic pancreatitis and celiac disease was negative. Fecal calprotectin levels were markedly elevated at 898 mcg/g (normal range: 0-120 mcg/g). A colonoscopy (Figure 1) revealed multiple punched-out ulcers distributed in a skip pattern (affected colon interspaced with normal appearing colon), and biopsies confirmed pancolitis.

**Figure 2:**
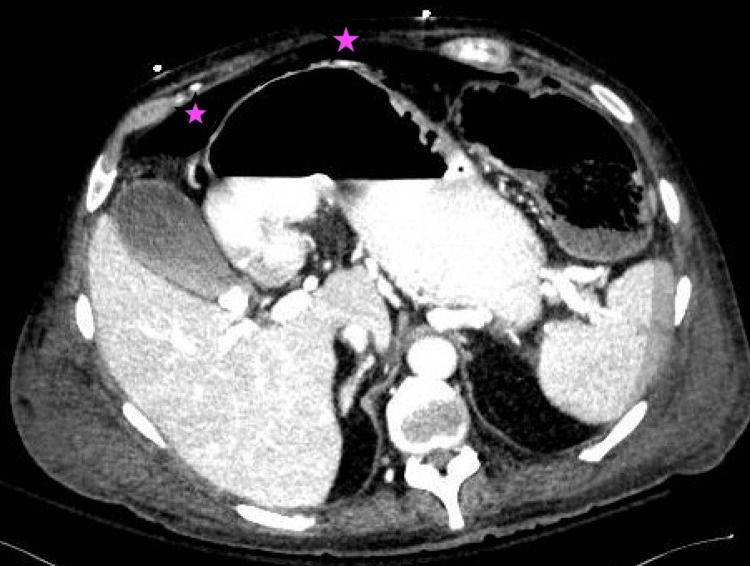
CT abdominal and pelvis. A large amount of pneumoperitoneum (marked star) without an exact free air source.

The patient's condition worsened, prompting an imaging study (Figure 2), which confirmed a large pneumoperitoneum secondary to bowel perforation that necessitated emergent surgical intervention. Intraoperative findings included multiple bowel perforations and a friable colon, which prompted a total colectomy with ileostomy.

**Figure 3:**
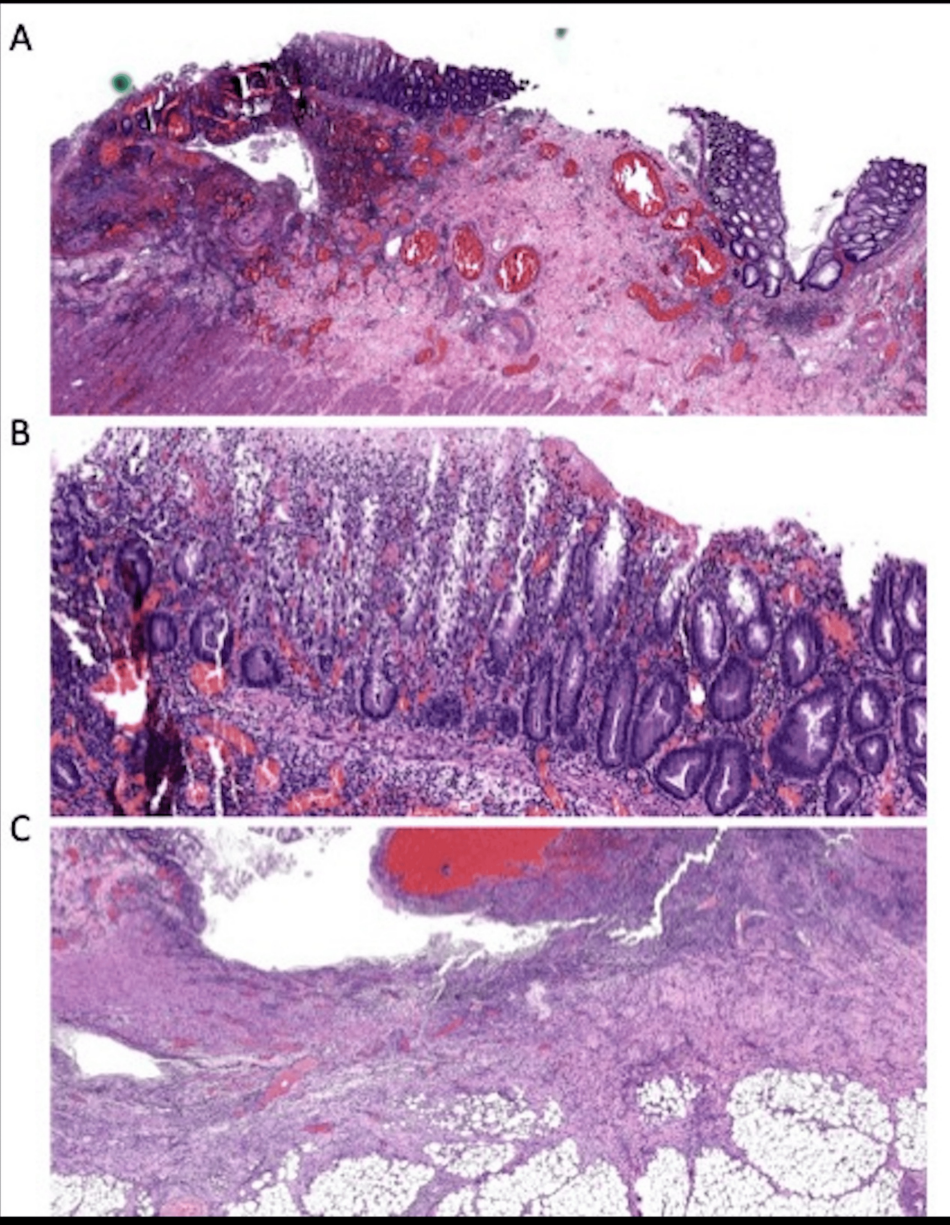
Surgical pathology micrograph of colon. Microscopic examination revealed extensive deep ulceration with multiple areas of fissuring, perforation, and serositis. Intramural bacterial aggregates were present focally. These findings are consistent with fulminant colitis, with most likely drug-related etiology.

Histopathological (Figure 3) examination revealed extensive deep ulceration with fissuring, perforation, and serositis areas. Intramural bacterial aggregates were also noted. These findings were consistent with fulminant colitis, likely related to MMF therapy. Unfortunately, the patient's condition was further complicated by polymicrobial bacteremia and progressive multi-organ failure, ultimately resulting in his death.

## Discussion

The exact mechanism underlying mycophenolate-induced colitis remains poorly understood; however, it is widely regarded as multifactorial. Key pathophysiological changes include significant alterations in the gut microbiome, which can disrupt the delicate balance of intestinal flora and compromise the gut's protective barrier. Additionally, mycophenolate inhibits purine synthesis in enterocytes, impairing cellular proliferation and repair mechanisms critical for maintaining mucosal integrity. Furthermore, the drug induces apoptosis of intraepithelial lymphocytes, which play a vital role in immune surveillance and mucosal defense. These combined effects lead to a weakened intestinal barrier, increased susceptibility to inflammation, and subsequent development of colitis. Calmet et al. analyzed solid organ transplant patients from 2000 to 2009, reporting a 9% incidence of mycophenolate mofetil (MMF) colitis [2]. Diarrhea was the most common symptom, present in 83% of cases. However, 47% of patients had normal colonoscopy findings, underscoring the importance of performing random biopsies when MMF colitis is strongly suspected. Histological findings varied widely: 50% showed acute colitis, 36% had inflammatory bowel disease (IBD) like features, 8.3 % resembled graft-versus-host disease (GVHD), and 5% appeared ischemic. Differentiating MMF colitis from GVHD is crucial, as graft-versus-host disease treatment involves intensifying immunosuppression, whereas MMF colitis management requires discontinuing mycophenolate mofetil.

Diagnosing mycophenolate mofetil (MMF) induced colitis can be challenging, as it requires ruling out other potential causes of chronic diarrhea in immunosuppressed patients. A colonoscopy with biopsy is essential to differentiate MMF-induced colitis from different etiology of colitis. However, a definitive diagnosis can be challenging, even with a biopsy. In our case, the biopsy revealed extensive ulceration with serositis while sparing the small bowel. Histological staining for cytomegalovirus (CMV) was negative. There are no universally accepted diagnostic criteria for MMF colitis. The clinical course and histological features can aid in diagnosis. Key features include Archbold et al. eosinophilia, the absence of endocrine cell aggregates in the lamina propria, and the lack of apoptotic microabscesses which is seen on our patient biopsy [3]. Mycophenolate mofetil has been successfully used as an immunosuppressive agent to treat IBD Tan et al. [4]. It is less likely that the patient would develop IBD after being on a stable dose for the past seven years. Based on the clinical presentation and biopsy findings, we believe the patient’s condition is consistent with MMF-induced colitis with fulminant features.

Farooqi et al. [1] and Alakkas et al. [5], most cases of MMF colitis (98%) resolve after discontinuing the drug; refractory cases may require additional treatments such as steroids or biologics Bouhbouh, et al. [6]. There are currently no standard guidelines for the management of mycophenolate-induced colitis. In our case, despite the withdrawal of MMF and aggressive management with intravenous steroids and infliximab, the patient developed fatal complications. This outcome underscores the severe risks associated with MMF-induced colitis. It highlights the importance of close monitoring and prompt intervention, including early proctocolectomy for patients suspected of fulminant mycophenolate mofetil (MMF) colitis.

## Conclusions

MMF-induced colitis is a rare but severe complication that can progress to life-threatening fulminant colitis. Early recognition and timely discontinuation of MMF are critical to improving outcomes. Although most cases improve with drug cessation, severe cases may require additional therapeutic interventions. This case emphasizes the importance of vigilant monitoring for gastrointestinal symptoms in patients on MMF, as delayed diagnosis and intervention can lead to devastating outcomes, including patient demise.
